# Be smart against cancer! A school-based program covering cancer-related risk behavior

**DOI:** 10.1186/1471-2458-14-392

**Published:** 2014-04-23

**Authors:** Friederike Stölzel, Nadja Seidel, Stefan Uhmann, Michael Baumann, Hendrik Berth, Jürgen Hoyer, Gerhard Ehninger

**Affiliations:** 1 University Cancer Center at the University Hospital Carl Gustav Carus Dresden, Fetscherstraße 74, 01307 Dresden, Germany; 2 TU Dresden, Department of Clinical Psychology and Psychotherapy, Hohe Straße 53, 01187 Dresden, Germany; 3 TU Dresden, Medical Faculty, Medical Psychology, Fetscherstraße 74, 01307 Dresden, Germany

**Keywords:** Adolescence, Cancer prevention, School-based health promotion, Health protective behavior, Outcome evaluation

## Abstract

**Background:**

Several studies suggest that most school-age children are poorly informed about cancer risk factors. This study examines the effectiveness of the ‘Be smart against cancer’ (BSAC) program in promoting cancer awareness and intentions to engage in health-promoting behavior.

**Methods:**

235 seventh-grade students were randomized to either the intervention (N = 152) or the wait-control group (N = 83). The intervention included the modules: “What is cancer?,” “Sun protection,” “Non smoking,” and “Physical activity, Healthy nutrition, and Limited alcohol consumption.” Outcomes measured at baseline and at the end of the one week BSAC program included knowledge of cancer and its behavioral risk factors, health-promoting intentions, and reported risk behavior.

**Results:**

BSAC was effective in increasing knowledge about cancer and risk factors for cancer (p < .001), as well as in increasing intentions to engage in health-promoting behavior (p < .001), independent of a student’s risk profile. Knowledge did not serve as a mediator for intention building.

**Conclusions:**

The BSAC is an effective school-based program for raising awareness of cancer, associated risk factors and intentions to engage in cancer-preventive behavior. The results indicate that the effectiveness of BSAC is independent of a student’s risk profile. Therefore, it holds considerable promise as a broadly applicable program to raise cancer awareness and promote healthy behavior intentions.

## Background

Although the overall incidence of cancer is rising, the World Health Organization (WHO) estimates that one third of all newly diagnosed cancers could be prevented if behavioral factors such as smoking, limited physical activity, unbalanced diet, alcohol consumption and excessive exposure to sunlight were changed [1-6].

Behaviors associated with increased cancer risk such as smoking and alcohol consumption emerge during childhood and adolescence [7-9] and once established, they contribute to cancer occurring later in life [10,11]. U.S. data from the Youth Risk Behavior Surveillance 2009 (YRBS) as well as data from the German Health Interview and Examination Survey for Children and Adolescents (KiGGS) and other European studies indicate that about 20% of high school students smoke cigarettes on a regular basis. An even higher proportion of students, 30% - 60%, drink alcohol regularly. Furthermore, 80% of high school students do not exercise regularly, and 10% are obese [12-16].

Different concepts of cancer education programs for adults which successfully increase knowledge and awareness have been reported [17-19]. Considering that changing extreme and stable risk behaviors is a difficult task, interventions would be most effective when directed at children and adolescents before they began to experiment with risk behavior [20,21]. Several studies suggest that a majority of school age children are poorly informed about cancer as well as preventive behavior [22-24]. The National Cancer Institute identified schools as having a central role in cancer prevention education [25]; school programs, however, often focus on only one behavioral risk factor [26-29]. We, therefore, designed the ‘Be smart against cancer’ (BSAC) program to target multiple risk factors for adult-onset cancer simultaneously by encouraging healthy lifestyles in a format especially designed for schools. In a one-week curriculum, the topics “What is cancer,” “Non smoking,” “Sun protection,” and “Physical activity, Healthy nutrition and Limited alcohol consumption” are covered in a practical manner for seventh-grade students. This age was chosen for the intervention because it is a common time for the initiation of risk behaviors [30,31], and these topics fit into the 7^th^ grade curriculum [32].

The aim of this study, which was run under the auspices of the University Cancer Center Dresden (UCC), a Comprehensive Cancer Center in the National Program of German Cancer Aid [33] was to promote the awareness of cancer-related risk factors and to increase the intention to engage in protective behavior as a predictor of actual behavior [34,35]. The effectiveness of BSAC in improving knowledge about cancer and associated behavioral risk factors and in promoting intentions to engage in protective behavior was tested in a randomized wait-control group trial including each student’s cancer risk profile as a variable in the analysis. Additionally, the capacity of knowledge gain as a mediator for a change of intentions was investigated.

## Methods

The study is based on an experimental pre-post design including an intervention group (IG) and a control group (CG). The IG received the BSAC curriculum during the school year 2008/2009 and the CG received no intervention. CG and IG were built on school-level. Effects were measured within a pre-post comparison of IG and CG. In the IG, the post-test was given at the last day of the project, and in the CG four to five days after the pre-test. BSAC was implemented in the CG the following school year without any further evaluation.

### Research participants

Since a low socio-economic status (SES) is linked to high levels of health-related risk behaviors [8,36,37], BSAC was implemented in vocationally-oriented secondary schools. Of vocational schools in Saxony (published online on http://www.schuldatenbank.sachsen.de), 20 were randomly selected using a random number table. Study personnel contacted the principals of the schools, inviting them to take part in the BSAC project. Eighteen agreed, of which three schools were assigned to the CG, again using a random number table.

**Figure 1:**
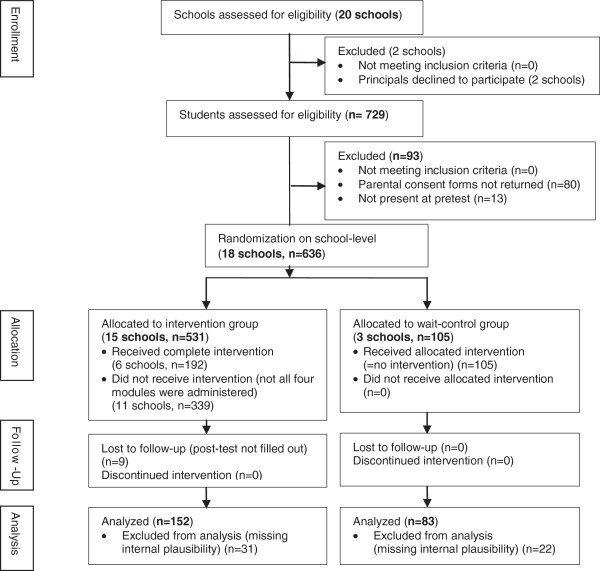
Modified CONSORT study flow diagram of study enrollment, allocation and analysis.

The school district classified BSAC as part of health education and the curriculum was delivered to all students in the IG. Thus, no consent for participation itself but for the evaluation was necessary. There were no exclusion criteria for students. Within the participating schools, consent forms regarding pre and post assessments were distributed to parents of all 729 seventh-grade students. A total of 636 (87%) gave informed consent for their children to be included in the study. In the IG some students did not receive the full BSAC curriculum, e.g. schools decided to participate only in two or three BSAC modules. A total of 152 IG and 83 CG students were eligible for analysis (29% of students allocated to the IG and 79% of students allocated to the CG, Figure 1) because only schools in which students completed all four modules were analyzed. A few students were excluded due to drop-out at post-test and a lack of internal plausibility (e.g. denying and admitting regular smoking habits in the same questionnaire). These 235 students (IG and CG) did not differ from students excluded from analysis (IG and CG, n = 401) with respect to the later defined risk-score (*t*(622) = 0.97, p = .33), knowledge-score (*t*(634) = 0.002, p = .998), intention-score (*t*(624) = 0.19, p = .85) at baseline and age (*t*(629) = − 0.46, p = .64), but did differ with respect to gender (included: 59% male, excluded: 48% male; *χ*^*2*^(1) *=* 7.87, p *=* .01)

### Instruments

To guarantee anonymity, all participating students used an individual four-figure code on each questionnaire for both the pre- and post-BSAC test. Due to the lack of an already existing instrument to assess knowledge about cancer and associated behavioral risk factors, the BSAC questionnaire was developed. Based on Danaei et al. [3], relevant behavioral risk factors were chosen and a set of items (actual behavior and behavioral intentions) was developed.

Experts in cancer and risk behavior revised the BSAC questionnaire, which was pilot-tested in 25 7^th^-grade students and modified thereafter. In addition to age and gender, the questionnaire contained questions regarding health-related behavior (BEH) in 6 areas: engaging in physical activity; applying sun lotion in the summer; smoking cigarettes; eating fruits and vegetables; eating sweets and drinking soft drinks; drinking alcohol, using a three or four-point response scale. There were also multiple-choice items with three response options testing knowledge reflecting the contents of the BSAC curriculum (KNO; 21 items), as well as intention to engage in protective behavior was assessed (INT; 6 items; eg: “in the next months, I intend to be physically active for at least 30 minutes,” “apply sun lotion,” “not smoke/smoke less,” “eat more fruits and vegetables,” “eat no/less sweets and drink no/less soft drinks,” and “drink no/less alcohol”), using a four-point response scale (Table 1).

**Table 1:** Examples for BSAC questionnaire items

**Topic**	**Item**	**Response scale**
**Smoking:**		
Behavior	Have you been smoking within the last three months?	not at all/1-2x per week/3-4x per week/(almost) daily
Knowledge	Second hand smoking means…	smoking another person’s cigarette/only infrequently smoking/inhaling the smoke of another person’s cigarette
Intention	I intend to smoke less/stop smoking.	not right/unlikely/likely/right
**Alcohol:**		
Behavior	Have you been drinking alcohol within the last three months?	not at all/1-2x per month/1-2x per week/more than twice a week
Knowledge	One should only drink alcohol…	seldom and in small quantities/only late at night/best with solid food
Intention	I intend to drink less/not at all.	not right/unlikely/likely/right
**Physical activity:**		
Behavior	How often are you physically active in your leisure time?	not at all/1-2x per week/more than 2x per week
Knowledge	How often should a person be physically active for at least 30 minutes?	3-4x per week/daily/2-3x per week
Intention	I intend to be physically active each day for at least 30 minutes.	not right/unlikely/likely/right
**Sun protection:**		
Behavior	Do you use sun lotion during the summer?	not at all/sometimes/always
Knowledge	What is an important factor for the development of skin cancer?	unclean skin/air pollution/UVA and UVB-radiation in the sunlight
Intention	I intend to use sun screen more often during the summer	not right/unlikely/likely/right

A risk-score was created based upon a combination of the six adolescent risk behaviors investigated [38]. Risk behaviors were dichotomized along guidelines for adolescent preventive care recommendations [39]. Participants received a value of ‘1’ for each risk behavior present (i.e. no sports or physical activity in leisure times, no usage of sun lotion in the summer, any rate of smoking within the last three months, consumption of fruits/vegetables less than twice a week, daily consumption of sweets or soft drinks, any consumption of alcohol within the last three months). A value of ‘0’ indicated absence of a certain risk behavior. Values were summed up into a risk-score ranging from ‘0’ (no risk behavior present) to ‘6’ (all risk behaviors present). Correct responses to the knowledge items were summed up into a knowledge-score with a range from ‘0’ to ‘21’. The intention-score was also created by summing responses (range ‘0’ to ‘18’). Retest-reliability of the final scales was tested on the CG, and was satisfactory (risk-score: r = .92, p < .001; knowledge-score: r = .75, p < .001; intention-score: r = .74, p < .001). Internal consistency was α = .37 for the risk-scale, α = .35 for the knowledge-scale and α = .62 for the intention-scale. Correlations between the risk-score and the knowledge-score (r = −.12, p = .07), between the risk-score and the intention-score (r = −.47, p < .001), and between the knowledge-score and the intention-score (r < .01, p = .97) indicated discriminant validity of the scales.

### Procedure

The BSAC curriculum is taught in one week and covers cancer and lifestyle factors associated with cancer risk reduction. The four modules are “What is cancer?,” “Non smoking,” “Sun protection,” and “Physical activity, Healthy nutrition and Limited alcohol consumption.” Each module requires one school day. A fifth day is used for recapitulating the project. All of the modules are organized similarly, with a short introduction, the specific content, and a rehearsal and summary of the module. Handouts are provided including the most important facts for the students.

The BSAC curriculum is based on the Theory of Planned Behavior [34]. All components and exercises are therefore designed to foster subjective norms, attitudes and perceived control over the actions concerning the specific preventive behaviors. Teaching materials were created by German Cancer Aid (Deutsche Krebshilfe e.V.), and were enhanced by medical and educational experts working at the UCC. A variety of didactic methods, such as information, class discussion, role-play, quiz, pantomime, group-work and video clips were used to enhance the social-cognitive prerequisites of intentions as well as the transmission of knowledge.

Concerning sun-protection, for instance, a class discussion about social norms (e.g. “My friends think being sun-tanned is pretty and healthy”) and attitudes (e.g. “I think sun-protection is important”) was conducted. A role-play supported perceived control over sun-protective behavior since practical training encourages the execution of actions. Students were informed about risks and use of the sun-light via a short video clip and tested on the benefits of different sun-protective behaviors via an interactive computer animation. Table 2 lists the topics and didactic methods used within each module.

**Table 2:** Topics of BSAC modules

**Module**	**Topic**	**Methods and content**
What is cancer?	Cancer development	Class discussion about student’s knowledge of cancer; information about cancer development on cell level
	Cancer treatment	Information on chemotherapy, radiation therapy, surgery
	Risk factors	Information about the ones capable of being influenced such as smoking and alcohol and the ones not capable of being influenced such as age
Sun protection	Positive and negative effects of UV-radiation	Information about Vitamin D production on one hand, elevated risk of skin damage and ultimately skin cancer on the other hand; discussion about these effects
	Skin types	Interactive animation: Students check their own skin type and their associated need for sun protection
	ABCD-Check for Melanoma	Information and training of ABCD-rules
	Sun protection guidelines	Short video and role-play: Information and trainings of sun protection; discussion about attitudes towards sun-protection
Non smoking	Effects of smoking on one’s health and other important facts about smoking	Information about effects of smoking; a class discussion about own/friend’s/families smoking experiences of each student and evaluation of its associated risks
	Reasons for non-smoking	Group work: Finding individual reasons for non-smoking, then adding further reasons out of a list together as a group
Physical activity, Healthy nutrition and Limited alcohol consumption	Physical activity	Short quiz: How long do we have to be physically active?
		Pantomime (3 students show forms of physical activity for the other students to guess): This is physical activity already?
		Handy exercises for in-classroom time: How can I be physically active in school?
	Healthy nutrition	The food pyramid for kids: Students fill in the different steps of the pyramid
		Classroom game: Right and wrong statements on nutrition
		Group-work: Healthy eating for a week – not difficult at all
		Short video on healthy nutrition: Summing it up
	Limited alcohol consumption	Information and discussion: Alcohol as risk factor for cancer

The BSAC teaching team consisted of members of the UCC and was trained by a teaching-experienced member of the UCC-Cancer Awareness Group. A manual provided all necessary facts about cancer and related risk factors and described the implementation of the curriculum.

### Data analysis

Group differences in continuous variables were assessed using t-Tests. Fisher’s exact test was used for dichotomous variables. One-way analysis of variance (ANOVA) was used to compare IG and CG at pre-test. Effects of the implemented curriculum regarding knowledge (pre-post difference) and intentions (pre-post difference) were tested using General linear mixed model (LMM) with experimental group (IG vs. CG) and risk-score (dichotomized into risk-score ≤ 1 vs. risk-score ≥ 2) as fixed effects, school as a random effect and pre-test scores as covariates. Pearsons r was calculated for the relationship between risk behavior, pre-test-scores and pre-post-differences of knowledge and intentions (IG sample only).

A mediation analysis (SOBEL, Version 3.6) to predict the intention to engage in protective behavior was applied with the experimental group as independent variable and knowledge as a mediator [40].

Differences in risk behavior at baseline between boys and girls of the IG were tested using a t-Test. Differences in knowledge gain and increase of intention were tested using LMM with gender and risk-score (dichotomized) as fixed effects, school as a random effect, and pre-test scores as covariates. Pearsons r was calculated for the relationship between age and risk behavior, gain in knowledge and increase of intention. Two-tailed tests were used and all statistics were performed using SPSS, Version 17.0 (SPSS Inc, Chicago, USA).

### Human subjects approval statement

The study was approved by the ethical board of the Technical University Dresden.

## Results

In total 235 students provided complete data at baseline. The students in both the IG and CG had a mean age of 13 years (range 12 to 15 years), and 41% of the participants were female. Table 3 shows significant negative relationships between risk-score and intentions to engage in health promoting behavior at pre-test, between pre-test scores and differences each in knowledge and intentions.

**Table 3:** Correlations (Pearsons r) of risk-score, pre-test scores and pre-post differences of knowledge and intention (IG-sample)

	**Risk-score**	**Knowledge-score (pre)**	**Intention-score (pre)**	**Difference knowledge**	**Difference intention**
Risk-score	1	-.12	-.47*	.08	.01
Knowledge-score (pre)	-	1	-.06	-.66*	.17
Intention-score (pre)	-	-	1	-.01	-.49*
Difference knowledge	-	-	-	1	-.07
Difference intention	-	-	-	-	1

*p < .01.

### Risk behavior

With regard to the six defined risk behaviors, 57% of the students in the CG and IG reported consumption of alcohol during the last three months, 25% stated (almost) daily consumption of sweets, and 14% reported not to be engaged in any physical activity in their leisure time (Table 4). Limited consumption of fruits and vegetables was reported by 17%, no application of sun lotion by 5%, and smoking by 6% of the students. Regarding the overall risk-score, 29% of the students showed a risk-score of zero, reporting no risk behavior at all. More than a third of the students (36%) reported one risk behavior only. No students reported five or more risk behaviors.

**Table 4:** Demographic characteristics, risk behavior, knowledge, and intentions across the Intervention group and control group participants

	**Total N = 235**	**CG**		**IG**		**Tests for significance**
		**n = 83**		**n = 152**		
		**CG pre**	CG post*	**IG pre**	IG post*	
**Demographic characteristics**						*Group difference*
Gender						χ^2^(1) = .74 p = 0.41
Male [n (%)]	139 (59)	46 (55)	**-**	93 (61)	**-**	
Female [n (%)]	96 (41)	37 (45)	**-**	59 (39)	**-**	
Age [mean (SD)]	13 (0.7)	13 (0.5)	**-**	13 (0.7)	**-**	t (1,219) = −0.91 p = .27
**Risk behavior** [n (%)]						*Group difference*
Physical inactivity	32 (14)	12 (15)	**-**	20 (13)	**-**	χ^2^(1) = 0.08 p = .84
No sun protection	11 (5)	3 (4)	**-**	8 (5)	**-**	χ^2^(1) = 0.33 p = .75
Smoking	15 (6)	7 (8)	**-**	8 (5)	**-**	χ^2^(1) = 0.88 p = .41
Insufficient intake of fruits/vegetables	41 (17)	18 (22)	**-**	23 (15)	**-**	χ^2^(1) = 1.60 p = .21
Daily intake of sweets	59 (25)	23 (28)	**-**	36 (24)	**-**	χ^2^(1) = 0.43 p = .53
Drinking alcohol	133 (57)	53 (65)	**-**	80 (53)	**-**	χ^2^(1) = 3.53 p = .07
**Risk score** [n (%)]						*Group difference*
0	66 (29)	20 (25)	**-**	46 (31)	**-**	
1	84 (36)	25 (31)	**-**	59 (39)	**-**	
2	50 (22)	23 (28)	**-**	27 (18)	**-**	
3	23 (10)	11 (14)	**-**	12 (8)	**-**	
4	8 (3)	2 (2)	**-**	6 (4)	**-**	
5	0	0	**-**	0	**-**	
6	0	0	**-**	0	**-**	
Mean (SD)	1.2 (1.1)	1.2 (1.1)	**-**	1.4 (1.1)	**-**	*F* (1,229) = 2.40 p = .12
**Knowledge** [mean (SD)]	-	13.4 (2.6)	13.2 (2.7)	12.8 (2.5)	17.5 (2.1)	*Group difference at pre-test F*(1,233) = 2.38 p = .12
						*Main effect: Experimental group F*(5,975) = 136.22 p < .001
						*Main effect: Risk behavior F*(212,696) = 0.09 p = .76
						*Interaction: Group x Risk behavior F*(212,745) = 2.03 p = .16
**Intention** [mean (SD)]	-	13.6 (3.2)	13.3 (3.6)	14.6 (2.5)	15.7 (2.5)	*Group difference at pre-test F*(1,229) = 4.14 p = .04
						*Main effect: Experimental group F*(5,772) = 16.93 p < .001
						*Main effect: Risk behavior F*(199,196) = 8.32 p < .01
						*Interaction: Group x Risk behavior F*(199,54) = 0.33 p = 0.57

*Regarding Demographic characteristics, Risk behavior and Risk-score only pretest data are analyzed.

Both CG and IG showed relatively low mean risk-scores which did not differ significantly between groups (p = .12). There was no significant difference between girls and boys regarding the risk-score (t (216) = 0.006, p = .995), but a significant correlation between age and risk-score (r = .19, p < .01).

### Knowledge

Knowledge about cancer and associated behavioral risk factors was significantly increased by the curriculum as compared to the control group (p < .001; Table 4). While there was a slight decrease in the CG, the knowledge-score increased in the IG. There was neither a significant main effect for the risk-score (p = .76) nor a significant interaction effect between experimental group and risk-score regarding the gain in knowledge (p = .16). Knowledge at pre-test was a significant covariate (p < .001).

Girls and boys in the IG did not differ significantly regarding the gain of knowledge (p = .97). As stated above, there was neither a significant main effect for the risk-score (p = .64) nor an interaction between gender and risk-score (p = .30), but knowledge at pre-test served as a significant covariate (p < .001). The correlation between age and the effectiveness regarding knowledge (r = −.04, p = .65) was not significant.

### Intention

At pre-test, the intention-score to engage in protective behavior differed between CG and IG (p = .04), showing a higher intention in the IG. Nevertheless, a significant intervention effect was observed for the intention-score (p < .01; Table 4) with an even higher level of intention for the IG at post-test. [41] There was a significant main effect for the risk-score (p < .01) but no significant interaction between experimental group and risk-score regarding the promotion of intentions. The intention-score at pre-test served as a significant covariate (p < .001).

Girls and boys in the IG did not differ significantly regarding the promotion of intention (p = .91). Again, there was a significant main effect for the risk-score (p < .01), but no interaction between gender and risk-score (p = .83). The intention-score at pre-test served as a significant covariate (p < .001). The correlation between age and effectiveness regarding intention (r = .03, p = .74) was not significant.

### Prediction of intention

The mediation analysis indicates a significant total effect of the experimental group on the promotion of intention (b (YX), p < .001), which is not mediated by the gain of knowledge (b (YM.X), p = .48), (Table 5).

**Table 5:** Mediation analysis on intention (pre-post difference)

	**Coeff**	**SE**	**Tests for significance**
b (YX)	−1.415	0.306	t = −3.978 p < .001
b (MX)	−4.634	0.340	t = −13.628 p < .001
b (YM.X)	−0.051	0.073	t = −0.704 p = .48
b (YX.M)	−1.652	0.490	t = −3.369 p < .001

X = experimental group, M = pre-post difference of knowledge-score, Y = pre-post difference of intention-score.

## Discussion

Certain risk behaviors established during adolescence, such as smoking, limited physical activity, unbalanced diet, alcohol consumption, and excessive exposure to sunlight, may have severe consequences in adulthood and may increase cancer risk [3]. Although health education has led to increased public awareness, the knowledge about some risk factors and their consequences has yet to be established among young adolescents [22,24]. Comparable to other programs, BSAC successfully implements knowledge about cancer and associated risk behaviors [17,18,23].

In addition to raising awareness, BSAC also effectively boosted health-promoting intentions. However, we found no mediating effect of knowledge on intentions. Other possible mediators such as social-cognitive determinants are discussed below. Since these variables have not been assessed, the discussion is speculative.

According to the Theory of Planned Behavior, intentions for behavior change are influenced by social-cognitive determinants such as subjective norms, attitudes and perceived behavior control [36]. As described above, these were addressed by the intervention. Other possible determinants of intention-building are described by the Health Action Process Approach (HAPA) [35] in which outcome expectancies and task self-efficacy play a crucial role in what people choose to do. Perceived risk may also stimulate intentions to adopt, initiate or maintain health behaviors. For Fischoff on the other hand, deciding to engage in health-promoting behavior depends on components as identifying alternative options and possible consequences [42]. All these variables may have been affected by the BSAC curriculum and some constructs might even overlap, for example perceived behavior control and self-efficacy. Thus, the role-play about sun-protection, designed to support perceived control and positive attitudes towards sun-protection, may also boost student’s self-efficacy to perform the desired behavior or outcome expectancies towards the judgment of peers. In summary, the mediating mechanisms of successfully fostered health-promoting intentions by BSAC remain hypothetical.

Good intentions cannot always be translated into corresponding actions, since various factors can be compromising [35]. As described in the HAPA-model, action planning, self-efficacy as well as situational determinants, e.g. barriers may play an important role. For adolescent smokers, intentions were found to predict planning. On the other hand, planning serves as a predictor for actual behavior. One possibility to foster self-efficacy and to overcome barriers in school settings might be the initiation of class-level projects. A contest of projects with students working on a specific cancer related risk behavior such as smoking is currently conducted by the UCC Prevention Center.

Almost one third of the participants reported no risk behavior at all and more than one third only one risk behavior. As described in other studies [11,43] there is also a significant number of students showing more than one risk behavior, thereby suggesting a co-occurrence of risk behaviors. Similar to the results of the KiGGS and the YRBS, alcohol consumption during the last three months was the most frequently reported risk behavior [14,15]. Alcohol consumption was followed by unhealthy eating habits and insufficient physical activity. However, only few students in this study engaged in risk behaviors, thus limiting the power of the program to reduce risk behaviors. The BSAC-program might therefore be successful at maintaining sufficient health behaviors rather than improving inadequate behaviors.

The role of actual risk in the process of changing intentions is controversial. Some studies suggest that risk behavior undermines the commitment to behavior change [44]. Data from the present study confirm a negative relationship between actual risk behavior and the willingness to engage in health-promoting behavior at baseline. Thus, students with a higher risk profile reported lower intentions to engage in health-promoting behavior. However, for students with low and high risk profiles, the program was equally able to raise the intentions as well as knowledge. These results suggest that the program is suitable for addressing both students with lower and higher risk profiles.

### Limitations

Due to practical issues, there was no randomization procedure on the individual-level. Experimental groups are based on natural groups (class-level). Therefore, the influence of confounding factors not assessed cannot be ruled out. Furthermore, self-reports are prone to several kinds of biases, such as social desirability [45]. No power calculation has been conducted beforehand; the authors conducted a post-hoc power calculation for the smallest interaction effect, resulting in a power of 0.8. The scales risk and knowledge shared a low internal consistency. The risk scale might suffer from a basement effect and knowledge and intention scales at post-test might show ceiling effects. One of the three schools in the control group did not complete the post-test on day five but on day four due to school internal reasons. Different measurement intervals bear the risk of influencing the observed outcomes. However, regarding risk-score, knowledge-score, and intention-score no differences between the four-day and the five-day measurement interval could be found. The study lacks a follow-up, which limits the explanatory power. Since the main focus of the curriculum was to promote awareness and intention, a change of behavior over the long term was not evaluated. Social-cognitive determinants of intention-building have not been assessed, thus limiting our ability to test for further mediating effects.

## Conclusions

The BSAC program was effective in raising awareness about cancer and its associated risk factors as well as health-promoting intentions. Since an integration of these topics into the official school curriculum is difficult, this project is one option to impart cancer knowledge in this age group. The results indicate that the effectiveness of BSAC is independent of the students’ risk profile and can therefore be implemented as a suitable program raising cancer awareness and health-promoting intentions.

## Abbreviations

ANOVA: Analysis of variance; BEH: Health-related behavior; BSAC: ‘Be smart against cancer’; CG: Control group; IG: Intervention group; INT: Intention to engage in health-protective behavior; KiGGS: German health interview and examination survey for children and adolescents; KNO: Cancer knowledge; LMM: General linear mixed model; SES: Socio-economic status; UCC: University cancer center dresden; YRBS: Youth risk behavior surveillance.

## Competing interests

The authors declare that they have no competing interests.

## Authors’ contributions

FS participated in the design and carried out the study. NS performed the statistical analysis. FS and NS drafted the manuscript. SU participated in the design and helped in the data analysis. MB, HB, JH and GE provided advice in the design of the study and contributed to the manuscript revision. GE is head of the project. All authors read and approved the final manuscript.

## Pre-publication history

The pre-publication history for this paper can be accessed here:

http://www.biomedcentral.com/1471-2458/14/392/prepub

## References

[B1] Boyle P Levin B World Cancer Report 2008 2008 International Agency for Research on Cancer: Lyon

[B2] Haberland J Bertz J Wolf U Ziese T Kurth B-M German cancer statistics 2004 BMC Cancer 2010 10 52 10.1186/1471-2407-10-52 20175882 PMC2837012

[B3] Danaei G Vander Hoorn S Lopez AD Murray CJL Ezzati M Causes of cancer in the world: comparative risk assessment of nine behavioural and environmental risk factors Lancet 2005 366 1784 1793 10.1016/S0140-6736(05)67725-2 16298215

[B4] Ullrich A Sepulveda C Yach D Straif K Beck V Public health und Krebsprävention: Initiativen der WHO - Perspektiven für Deutschland Der Onkol 2004 10 166 174 10.1007/s00761-003-0626-7

[B5] Department of Health The NHS Cancer Plan: A Plan for Investment, a Plan for Reform 2000 London: DOH

[B6] World Health Organisation Strategies to Improve and Strengthen Cancer Control Programmes in Europe 2003 Geneva: Report of a WHO Consultation, WHO

[B7] Nickel J Ravens-Sieberer U Richter M Settertobulte W Richter M, Hurrelmann K, Klocke A, Melzer W, Ravens-Sieberer U Gesundheitsrelevantes Verhalten und soziale Ungleichheit bei Kindern und Jugendlichen Gesundheit, Ungleichheit und Jugendliche Lebenswelten: Ergebnisse der zweiten internationalen Vergleichsstudie im Auftrag der Weltgesundheitsorganisation WHO 2008 Weinheim: Juventa 63 92

[B8] Hähne C Dümmler K Richter M, Hurrelmann K, Klocke A, Melzer W, Ravens-Sieberer U Einflüsse von Geschlecht und sozialer Ungleichheit auf die Wahrnehmung und den Umgang mit dem Körper im Jugendalter Gesundheit, Ungleichheit und Jugendliche Lebenswelten: Ergebnisse der zweiten internationalen Vergleichsstudie im Auftrag der Weltgesundheitsorganisation WHO 2008 Weinheim: Juventa 93 113

[B9] DiClemente RJ (Ed): Handbook of Adolescent Health Risk Behavior 1996 New York: Plenum Press

[B10] Windle M Grunbaum JA Elliott M Tortolero SR Berry S Gilliland J Kanouse DE Parcel GS Wallander J Kelder S Collins J Kolbe L Schuster M Healthy passages. A multilevel, multimethod longitudinal study of adolescent health Am J Prev Med 2004 27 164 172 10.1016/j.amepre.2004.04.007 15261905

[B11] Weissberg RP Kumpfer KL Seligman MEP Prevention that works for children and youth: an introduction Am Psychol 2003 58 425 432 12971188 10.1037/0003-066X.58.6-7.425

[B12] Kurth B-M Schaffrath Rosario A The prevalence of overweight and obese children and adolescents living in Germany. Results of the German Health Interview and Examination Survey for Children and Adolescents (KiGGS) Bundesgesundheitsblatt Gesundheitsforschung Gesundheitsschutz 2007 50 736 743 10.1007/s00103-007-0235-5 17514458

[B13] Lampert T Mensink GBM Romahn N Woll A Physical activity among children and adolescents in Germany. Results of the German Health Interview and Examination Survey for Children and Adolescents (KiGGS) Bundesgesundheitsblatt Gesundheitsforschung Gesundheitsschutz 2007 50 219 226 10.1007/s00103-007-0224-8 17514447

[B14] Lampert T Thamm M Tabak-, Alkohol- und Drogenkonsum von Jugendlichen in Deutschland Bundesgesundheitsblatt Gesundheitsforschung Gesundheitsschutz 2007 50 600 608 10.1007/s00103-007-0221-y 17514444

[B15] Eaton DK Kann L Kinchen S Shanklin S Ross J Hawkins J Harris W Lowry R McManus T Chyen D Lim C Whittle L Brener N Wechsler H Youth Risk Behavior Surveillance - United States, 2009 Morb Mortal Wkly Rep Youth Risk Behav Surveil 2010 59 1 142 20520591

[B16] Martinez-Gomez D Ortega FB Ruiz JR Vicente-Rodriguez G Veiga OL Widhalm K Manios Y Béghin L Valtueña J Kafatos A Molnar D Moreno LA Marcos A Castillo MJ Sjöström M Excessive sedentary time and low cardiorespiratory fitness in European adolescents: the HELENA study Arch Dis Child 2011 96 240 246 10.1136/adc.2010.187161 21220264

[B17] Robb KA Miles A Campbell J Evans P Wardle J Can cancer risk information raise awareness without increasing anxiety? A randomized trial Prev Med 2006 43 187 190 10.1016/j.ypmed.2006.04.015 16765428

[B18] Wolf RL Lepore SJ Vandergrift JL Basch CE Yaroch AL Tailored telephone education to promote awareness and adoption of fruit and vegetable recommendations among urban and mostly immigrant black men: a randomized controlled trial Prev Med 2009 48 32 38 10.1016/j.ypmed.2008.10.015 19010349 PMC4537646

[B19] Baum A Cohen L Successful behavioral interventions to prevent cancer: the example of skin cancer Annu Rev Publ Health 1998 19 319 333 10.1146/annurev.publhealth.19.1.319 9611622

[B20] Gerrard M Gibbons FX Benthin AC Hessling RM A longitudinal study of the reciprocal nature of risk behaviors and cognitions in adolescents: what you do shapes what you think, and vice versa Heal Psychol 1996 15 344 354 10.1037//0278-6133.15.5.344 8891713

[B21] Soldz S Kreiner P Clark TW Krakow M Tobacco use among Massachusetts youth: is tobacco control working? Prev Med 2000 31 287 295 10.1006/pmed.2000.0727 11006052

[B22] Divakaran B Muttapillymyalil J Sreedharan J Shalini K Lifestyle riskfactors of noncommunicable diseases: awareness among school children Indian J Cancer 2010 47 Suppl 1 9 13 20622407 10.4103/0019-509X.63864

[B23] Schonfeld DJ Bases H Quackenbush M Mayne S Morra M Cicchetti D Pilot-testing a cancer education curriculum for grades K-6 J Sch Health 2001 71 61 65 10.1111/j.1746-1561.2001.tb06492.x 11247381

[B24] Mahdy N Fatohy I Assessment of students’ knowledge, attitude and practice concerning cancer and its prevention. Part I J Egypt Public Health Assoc 1998 73 399 431 17219931

[B25] D’Onofrio CN Making the case for cancer prevention in the schools J Sch Health 1989 59 225 231 10.1111/j.1746-1561.1989.tb04710.x 2739366

[B26] Balyaci OE Kostu N Temel AB Training program to raise consciousness among adolescents for protection against skin cancer through performance of skin self examination Asian Pac J Cancer Prev 2012 13 5011 5017 10.7314/APJCP.2012.13.10.5011 23244101

[B27] Dobbins M De Corby K Robeson P Husson H Tirilis D School-based physical activity programs for promoting physical activity and fitness in children and adolescents aged 6–18 Cochrane Database Syst Rev 2009 Issue 1 CD007651 10.1002/14651858.CD007651 19160341

[B28] Thomas RE McLellan J Perera R School-based programmes for preventing smoking Cochrane Database Syst Rev 2013 Issue 4 CD001293 10.1002/14651858.CD001293.pub3 PMC7028068 23633306

[B29] Buller DB Reynolds KD Yaroch A Cutter GR Hines JM Geno CR Maloy JA Brown M Woodall WG Grandpre J Effects of the Sunny Days, Healthy Ways curriculum on students in grades 6 to 8 Am J Prev Med 2006 30 13 22 10.1016/j.amepre.2005.08.046 16414419 PMC1448611

[B30] Tercyak KP Tyc VL Opportunities and challenges in the prevention and control of cancer and other chronic diseases: children’s diet and nutrition and weight and physical activity J Pediatr Psychol 2006 31 750 763 16820383 10.1093/jpepsy/jsj126

[B31] Michels KB The role of nutrition in cancer development and prevention Int J Cancer 2005 114 163 165 10.1002/ijc.20662 15540221

[B32] Sächsisches Ministerium für Kultur und Sport Lehrplan Mittelschule 2009 Dresden: Juventa

[B33] Richter M Gesundheit Und Gesundheitsverhalten Im Jugendalter: Der Einfluss Sozialer Ungleichheit 2005 Wiesbaden: VS

[B34] Ravens-Sieberer U Erhart M Richter M, Hurrelmann K, Klocke A, Melzer W, Ravens-Sieberer U Die Beziehung zwischen sozialer Ungleichheit und Gesundheit im Kindes- und Jugendalter Gesundheit, Ungleichheit und Jugendliche Lebenswelten: Ergebnisse der zweiten internationalen Vergleichsstudie im Auftrag der Weltgesundheitsorganisation WHO 2008 Weinheim: Juventa 38 62

[B35] Baumann M Ehninger G Herrmann T Saeger H-D Simon M Etablierung eines interdisziplinären Krebszentrums im Spannungsfeld interner und externer Interessen Zeitschrift für ärztliche Fortbildung und Qual im Gesundheitswes 2007 101 153 158 10.1016/j.zgesun.2007.02.004 17608032

[B36] Ajzen I The theory of planned behavior Organ Behav Hum Decis Process 1991 50 179 211 10.1016/0749-5978(91)90020-T

[B37] Schwarzer R Luszczynska A How to Overcome Health-Compromising Behaviors Eur Psychol 2008 13 141 151 10.1027/1016-9040.13.2.141

[B38] Tercyak KP Abraham AA Graham AL Wilson LD Walker LR Association of multiple behavioral risk factors with adolescents’ willingness to engage in eHealth promotion J Pediatr Psychol 2009 34 457 469 10.1093/jpepsy/jsn085 18723566 PMC2684486

[B39] Richmond TK Freed GL Clark SJ Cabana MD Guidelines for adolescent well care: is there consensus? Curr Opin Pediatr 2006 18 365 370 10.1097/01.mop.0000236383.41531.8e 16914988

[B40] Preacher KJ Hayes AF SPSS and SAS procedures for estimating indirect effects in simple mediation models Behav Res Methods Instrum Comput 2004 36 717 731 10.3758/BF03206553 15641418

[B41] Cohen J Statistical Power Analysis for the Behavioral Sciences 1988 2 New Jersey: Lawrence Erlbaum Associates

[B42] Igra V Irvin CE DiClimente RJ, Hansen WB, Ponton LE Theories of Adolsecent Risk-Taking Behavior Handbook of Adolescent Health Risk Behavior 1996 New York: Plenum Press 35 52

[B43] Tercyak KP Donze JR Prahlad S Mosher RB Shad AT Multiple Behavioral Risk Factors Among Adolescent Survivors of Childhood Cancer in the Survivor Health and Resilience Education (SHARE) Program Pediatr Blood Cancer 2006 47 May 2005 825 830 16333821 10.1002/pbc.20602

[B44] Longshore D Stein JA Conner BT Psychosocial antecedents of injection risk reduction: a multivariate analysis AIDS Educ Prev 2004 16 353 366 10.1521/aeap.16.4.353.40395 15342337

[B45] Morbitzer P Spröber N Wie zuverlässig sind Selbsteinschätzungen von Schülern zum Vorkommen von Bullying? Prax Kinderpsychol Kinderpsychiatr 2009 58 81 95 19334399 10.13109/prkk.2009.58.2.81

